# Something Is Missing: Addressing Racial Diversity, Equity, Inclusion and Representation in Canadian Gastroenterology

**DOI:** 10.1093/jcag/gwab039

**Published:** 2021-10-08

**Authors:** Chadwick I Williams

**Affiliations:** 1 Department of Internal Medicine, Dalhousie University, Halifax, Nova Scotia, Canada; 2 Department of Internal Medicine, Dartmouth General Hospital, Dartmouth, Nova Scotia, Canada

Incorporating equity, diversity and inclusion (EDI) has become a more prominent focus of the medical institution over the last 2 years. This has been sparked by a wider, global awareness and by a movement to recognize and address systemic racism, with a goal to end social and racial injustice.

Gastroenterology (GI) in Canada remains a field of predominantly White, cis-gender, male physicians. While Canadian data on diversity in GI are lacking, Bollegala et al demonstrated that women are under-represented in resident training programs and are nearly absent from key leadership roles within our GI programs and organizations (1). As we acknowledge this important disparity, we cannot help but to notice that something else is also glaringly absent.

As Canadian society grows more diverse, the rather homogenous sub-specialty of GI becomes blatantly less reflective and representative of the general population. African Canadians and First Nations peoples are notably under-represented in GI. I am a Black gastroenterologist and I have been practising for 10 years. I attend and participate in multiple conferences and research gatherings yearly. I know of only two other Black gastroenterologists practicing in all of Canada! I also know of only one gastroenterologist who identifies as First Nations! As Canadian gastroenterologists we need to ask ourselves some important questions. Why are these groups so woefully under-represented in our specialty? How do we resolve this issue?

Canadian gastroenterology needs to do much better. Improved racial diversity and representation in gastroenterology is necessary for several reasons. More diverse medical teams function better (2). They foster better perspective and more varied experiences and allow for improved rapport and understanding of the medical needs and cultural nuances of patients. BIPOC (Black, Indigenous and other people of color) physicians are also more likely to consider practicing in BIPOC communities. Such communities are historically under-serviced. Data have shown that racism is an independent determinant of health outcomes (3,4). Unfortunately, in Canada, race-based data are not readily captured, making it even more difficult to ascertain the true severity of this problem. A glance at the demographic tables from most of our clinical trials in gastroenterology, reveals that racialized groups are not well represented in these studies at all.

Improved diversity is an important way to address race-based health care disparities. Racial bias, whether explicit or implicit, affects our decision making at multiple levels including medical school admissions boards (5), GI training program selections, and faculty promotion and even remuneration. Working diligently to identify these biases and to neutralize them is an important step toward improved diversity in gastroenterology in Canada. Diversity alone is not enough though. BIPOC gastroenterologists and trainees need to have equity and inclusion also. They need to be included, empowered and heard; not simply integrated or incorporated into the world of GI.

It is heartening that Canadian Association of Gastroenterology (CAG) has identified EDI as an important issue. It is a complex and multi-faceted problem and requires a well-planned approach. Fortunately, much work has already been done in other jurisdictions and CAG can build on these learnings and approaches. The American Association of Gastroenterology has an Equity Task Force that was developed from their Diversity Committee that was established in 1993 (6). The task force emphasizes the need for action at several levels including: workforce and leadership, recognition of BIPOC contributions to GI and medicine and management of biases, research and funding, justice and equity and education. We can build on this and utilize a similar multi-pronged approach to address EDI in Canada.

In Canada, most medical schools are taking measures to address the effect of racial bias and to improve equity and diversity in their programs. This of course must involve more than simply increasing the percentage of BIPOC students. These institutions must also look objectively at their own internal structures, boards, and policies. Our GI training programs, and fellowship programs must do the same. CAG has developed a Diversity and Equity committee and the organization’s revered GI Scholars’ Program has taken this issue very seriously and has added two additional seats to address a lack of diversity and to give more BIPOC trainees exposure and opportunities consider careers in gastroenterology and hepatology. The fact is that the status quo is designed, it is old, and it is inequitable. It is the product of systemic racism, and subsequent institutionalized norms and processes that promote it. We as Canadian gastroenterologists need to recognize this and commit to change so that we can develop stronger, more functional teams and provide better, more culturally competent care to our patients.
